# Peri-Operative Optimization of Patients with Crohn’s Disease

**DOI:** 10.1007/s11894-024-00925-9

**Published:** 2024-02-29

**Authors:** Hareem Syed, Ahmed Nadeem, David Gardinier, Kendra Weekley, Dovid Ribakow, Stephen Lupe, Shubha Bhat, Stefan Holubar, Benjamin L. Cohen

**Affiliations:** 1 Department of Internal Medicine, Cleveland Clinic Foundation, 9500 Euclid Avenue, Cleveland, OH 44195 USA; 2 Digestive Disease and Surgery Institute, Cleveland Clinic Foundation, 9500 Euclid Avenue, Cleveland, OH 44195 USA

**Keywords:** Crohn’s disease, Peri-operative, Nutrition, Immunosuppression, Intra-abdominal abscesses

## Abstract

**Purpose of Review:**

The management of patients with Crohn’s disease (CD) undergoing surgery is complex and optimization of modifiable factors perioperatively can improve outcomes. This review focuses on the perioperative management of CD patients undergoing surgery, emphasizing the need for a multi-disciplinary approach.

**Recent Findings:**

Research highlights the benefits of a comprehensive strategy, involving nutritional optimization, psychological assessment, and addressing septic complications before surgery. Despite many CD patients being on immune-suppressing medications, studies indicate that most of these medications are safe to use and should not delay surgery. However, a personalized approach for each case is needed.

**Summary:**

This review underscores the importance of multi-disciplinary team led peri-operative management of CD patients. We suggest that this can be done at a dedicated perioperative clinic for prehabilitation, with the potential to enhance outcomes for CD patients undergoing surgery.

## Introduction

Crohn’s disease (CD), a chronic inflammatory disease of the gastrointestinal tract, is rising in incidence worldwide [1]. Population-based studies have shown that with advances in medical treatment and the increasing use of biologics, the rate of surgery has declined. However, even in the contemporary era, up to one-third of patients with CD require major abdominal surgery within five years of diagnosis [2, 3].

Although generally safe, surgery in patients with CD is associated with a higher rate of complications than in other patients undergoing abdominal surgery. Commonly reported complications include wound infections, anastomotic leaks, abscesses, sepsis, and non-surgical site infections [4]. Older age, emergent or urgent surgery, and experience of the surgeon and facility are non-modifiable factors associated with increased postoperative complications and morbidity [5, 6]. Other, potentially modifiable risk factors include the use of preoperative immunosuppressive medications (e.g., corticosteroids), malnutrition, anemia, smoking, and intra-abdominal sepsis [7].

Although many of these risk factors have been well studied, there is a lack of research on preoperative optimization for patients with CD. In this review, we aim to describe modifiable risk factors for postoperative complications in patients with CD and discuss strategies to improve surgical outcomes.

## Patient Communication, Engagement, and Multidisciplinary Planning

A significant proportion of CD patients undergo surgery throughout their lifetime; however, most view surgery negatively, often believing that surgery is a last resort after failure of medical management rather than a useful treatment option [8]. This negative perception may lead to a delay in surgery and be associated with worse outcomes, as surgery may occur under urgent/emergent rather than elective circumstances. Therefore, it is imperative that all physicians involved in the care of patients with CD frame surgery as a therapeutic option and not a failure of therapy or something to be avoided, lest it be positioned “too late” and set the stage for increased risk of postoperative complications.

An international survey of 425 patients with CD showed that most patients primarily received information about surgery from their gastroenterologists, and surgeons were often not involved until later in the disease course. A possible result is that up to 1/3rd of patients felt that the indication for surgery was not a shared decision [8]. It is important for patients undergoing surgery to be well informed about all aspects of their care, including the indications for surgery, risks and benefits of surgery, and expectations of life after surgery.

Patients with IBD have rated the concern over having an ostomy bag or undergoing surgery as the top concern, which persists even when the symptoms improve; therefore, the role of a stoma nurse in the team is invaluable [9]. Many CD patients have altered body image and decreased self-esteem related to their bowel habits, which may be exacerbated by an ostomy; therefore, comprehensive stoma counselling and setting expectations for recovery have been found to mitigate the patient’s anxiety and adequately prepare them for optimal living and functionality post-surgery [10].

It is well known that patients with IBD have higher rates of anxiety and depression than the general population which can worsen in the perioperative period due to heightened anxiety and fear related to surgery [11, 12]. In addition, both symptoms of depression and anxiety have been correlated with increased pain perceptions and decreased physiological outcomes post-surgery. Furthermore, the development of trauma related to treatment has been found to be increased in patients with Crohn’s disease and symptoms of trauma have been related to undergoing surgery in this population [13, 14]. It is therefore important to have a psychologist on the multi-disciplinary team (MDT) to help recognize and aide in managing the patient’s mental and behavioral health concerns early on as it can have an impact not just on their quality of life (QOL) but also on their disease and surgical course [15].

Patients undergoing surgery for CD should be evaluated and treated by an MDT including a surgeon, gastroenterologist, nutritionist, stoma nurse, and psychologist. Although not always patient facing, engaged pathologists and radiologists are also a critical part of the MDT. A Chinese study exploring the role of an MDT in management of IBD patients showed decreased rates of postoperative clinical recurrence and subsequent surgery, decreased frequency of emergency surgery, and improved preoperative nutritional status [16]. Similarly, a retrospective analysis of UC patients undergoing surgery showed that patients who received care after the implementation of an MDT bundle had lower rates of postoperative complications and better outcomes than those who received care before implementation of an MDT bundle [17]. Although real world data is limited, we know that surgery for Crohn’s is complex and requires integrated care with multiple specialties, shared and informed decision making with the patient, and extensive counselling to prepare the patient for success.

## Preoperative Imaging and Endoscopy

Cross-sectional imaging and endoscopy before surgery are crucial for disease staging and provide a roadmap for successful surgery. In cases where segmental colon or small bowel resections are planned, disease activity assessments from both cross-sectional imaging and endoscopy can help define the severity of illness and extent of resection and are critical for planning interventions such as bowel-preserving techniques (strictureplasties) and diversion. In addition, imaging may distinguish between ongoing active inflammation and fibrosis. Patients with significant pre-stenotic dilation (> 3 cm) are less likely to respond to medical therapy and may benefit from timely surgery [18]. Other ancillary information that would change management include the presence of significant inflammatory changes in the adjacent mesentery with hydronephrosis, the need for ureteral stenting perioperatively, and extraluminal penetrating complications, requiring additional measures such as percutaneous drainage of abscesses and resection of other segments of the bowel involved in fistulae [19].

Computed tomography enterography (CTE) and magnetic resonance enterography (MRE) provide high-quality imaging for the evaluation of intraluminal and extraluminal CD and are the recommended standards of care for perioperative imaging. Large meta-analyses and prospective studies have shown that both CTE and MRE are equally sensitive, in the range of 90%, in assessing CD disease activity and distinguishing active inflammation, strictures, and abscesses [20–22]. Hence, the choice of imaging modality should be based on the risk of exposure and accessibility of either modality. Modern CT protocols typically use lower radiation doses, while MRE obviates radiation exposure but may induce claustrophobia and, in general, are less readily available.

## Management of Intra-abdominal Septic Complications (IASC)

Intra-abdominal abscesses are common and develop in 10–30% of patients with CD during the course of their disease [23, 24]. The presence of an abscess at the time of surgery is associated with an increased risk of need for a diverting stoma and postoperative intra-abdominal septic complication (IASC) such as abscesses and anastomotic leaks [25–27]. 

Commonly used treatments for abscesses include medical management, percutaneous imaging-guided drainage, or immediate surgical drainage. A meta-analysis of nine studies showed a three times higher chance of achieving abscess resolution with surgical management than medical therapy alone [28]. Although surgery remains the definitive treatment for IASC, preoperative percutaneous drainage (PD) of abscesses has been shown to decrease the rate of postoperative complications and the need for stoma. A meta-analysis comparing patients with PD followed by either no surgery or as a bridge to surgery with those who underwent surgery directly found that patients in the PD group had decreased rates of overall complications and postoperative abscess recurrence [29]. This initial nonoperative approach may delay the need for surgery and allow for medical and nutritional therapy to optimize the patient for surgery. Although the adequate time to wait for surgery after PD has not been established, a retrospective multicenter study suggested that an interval of 2–4 weeks is safe and associated with a low risk of abscess recurrence and stoma formation [30]. The management of abscesses in CD is challenging and ideally performed by MDT using tailored approaches for each individual case.

## Preoperative Nutritional Status

The American Society of Parenteral and Enteral Nutrition (ASPEN) defines malnutrition as any nutritional imbalance [31]. Malnutrition is common in patients with IBD, with an incidence of 20–85% in patients with CD and a significantly higher incidence in hospitalized patients with IBD than in non-IBD patients [32, 33]. Malnutrition in IBD develops secondary to reduced oral intake, increased nutrient requirements, increased nutrient loss, and occasional drug interactions, such as dysgeusia (metronidazole) and anorexia (penicillins). Patients with CD may remain at an elevated risk of malnutrition even when their disease is quiescent [33, 34]. Therefore, it is important to screen all patients with CD for malnutrition regardless of their disease activity.

Preoperative malnutrition is an independent risk factor for postoperative complications including IASC [35]. Markers to identify at-risk patients include low body mass index (BMI), unintentional weight loss exceeding 10% of total body weight, reduced anthropometry or grip strength, and low albumin levels. Patients with BMI < 17.5 kg/m^2^ have 7.35 times higher risk of developing postoperative infectious complications [36]. Similarly, patients with preoperative weight loss of > 10% of body weight are twice as likely to develop IASC postoperatively [37].

It is important to note that although hypoalbuminemia is common in patients with IBD [38] and is often used as a marker for malnutrition, it does not reflect total body protein or muscle mass and should not be used as a marker of nutrition [39]. Preoperative hypoalbuminemia may also be reflective of systemic inflammation as both albumin and prealbumin are negative acute phase reactants and their concentrations decline in the presence of inflammation. Although not reflective of nutritional status, the presence of low albumin is associated with increased post-operative complications even in patients with a normal BMI [40].

Preoperative nutritional optimization of patients diagnosed with malnutrition, either in the form of enteral nutrition (EN) or total parenteral nutrition (TPN), may reduce the risk of postoperative complications by up to 74% [41]. Exclusive enteral nutrition (EEN) has been shown to induce mucosal healing and remission and has been linked to decreased CRP levels, increased BMI, attenuation of anemia, and significantly lower infectious and noninfectious complications after surgery [42]. While EEN is typically provided for a period of 6–12 weeks to induce remission, EEN for as little as 4 weeks pre-operatively is associated with improved outcomes [40, 43]. The use of TPN over EEN is not generally recommended as it does not result in better outcomes while carrying an infectious and thrombotic risk. However, TPN may be necessary in certain situations, such as intestinal obstruction or ileus, severe shock, intestinal ischemia, high-output fistula, or severe intestinal hemorrhage, where there is a high risk of EN failure.

Malnutrition should be identified early, and a dietician should be part of MDT care for patients with CD. No single diet fits all IBD patients, and each patient requires careful evaluation and optimization in accordance with their specific protein and caloric requirements, dietary intolerance, and food preferences. Although patients with IBD do not have increased caloric requirements at baseline, patients with active disease are known to have increased protein requirements and should receive 1.2–1.5 g/kg/d of protein in their diet [44]. Additionally, all patients should be screened for micronutrient deficiencies and the presence of other malabsorption syndromes such as lactose intolerance or celiac disease (Table 1).

**Table 1:** Perioperative nutritional optimization

Screen	o Malnutrition: Weight loss of more than 10–15% in 6 months or BMI < 18.5 o Anemia o Hypoalbuminemia o Micronutrient deficiency: Iron, Zinc, Vitamin D o Malabsorption syndromes: Celiac disease, lactose intolerance
Initiate Nutrition Therapy	o Referral to IBD dietitian for nutrition counseling o If malnutrition identified: Delay elective IBD surgery until intensive artificial feeding initiated o Correct anemia and any micronutrient deficiencies o EEN for 4 weeks preferred for patients with malnutrition, stricture, or fistula o Parenteral nutrition only if enteral nutrition is contraindicated o Stricture: EEN or adapted texture diet preferred, NPO or TPN only for active obstruction
Post operative	o Initiate early enteral nutrition (within 24 h of surgery as it is associated with improved outcomes) o Re-assessment of nutrition status with referral to IBD dietitian if patient is at risk for malnutrition [96].

Patients undergoing elective surgeries should be treated according to the enhanced recovery protocol (ERP) guidelines, which include maintaining perioperative homeostasis by avoiding prolonged preoperative fasting, re-establishing oral feeding as early as possible, limiting the use of drains and catheters, employing minimally invasive (laparoscopic) surgery liberally, early mobilization, glucose control, and integration of nutrition into the overall management of the patient [44]. Whenever possible, elective surgery should be delayed 7–14 days in patients diagnosed with severe malnutrition to optimize nutrition prior to surgery. In the case of emergency surgeries and severe protein-calorie malnutrition, EN or TPN should ideally be started within 24 h postoperatively [44] .

## Anemia

Anemia is prevalent in IBD, with rates varying from 6 to 74%, and is usually a combination of chronic anemia and iron deficiency anemia [45]. In the preoperative setting, patients with anemia are more likely to develop preoperative sepsis and require emergency surgery. They were also more likely to have prolonged hospital stays, more postoperative complications, and higher reoperation rates [46]. Therefore, it is important to identify and manage anemia in patients undergoing surgery. Patients with mild anemia and inactive disease can be treated with oral iron supplements; however, studies have shown that parenteral iron is more effective, faster, and better tolerated than oral iron, which is associated with additional gastrointestinal discomfort, constipation, and dark stools. Hence, parenteral therapy should be considered the first-line treatment for patients with active disease and anemia [47]. 

## Smoking Cessation

Cigarette smoking is a risk factor for many perioperative complications including surgical site infections and postoperative IASC in patients with CD [48]. Current smokers are 50% more likely to develop surgical site infections (SSI). Interestingly, smoking on the day of surgery was independently associated with the development of SSI (OR 1.96, CI, 1.20–1.90) suggesting that abstinence even on the day of surgery could reduce the risk of complications [49]. 

Additionally, patients with CD who smoke have a 2.5X increased risk of surgical recurrence compared with non-smokers [50]. Therefore, it is important to counsel patients to quit smoking prior to surgery in CD not only to prevent postoperative complications but also to reduce the risk of surgical recurrence. A randomized controlled trial of a smoking cessation intervention program, including counselling and nicotine replacement therapy, 6–8 weeks before elective orthopedic surgery, significantly reduced postoperative morbidity [51]. We recommend referral to a smoking cessation clinic for all patients with CD who are scheduled for elective surgery. In patients who do not have enough time to go through a formal program, counselling to stop smoking and abstain from smoking on the day of surgery can also prove helpful.

## Venous Thromboembolism Prophylaxis

Patients with IBD have a two to three fold increased risk of venous thromboembolism (VTE) compared with the general population [52, 53]. This risk is higher during active disease and hospitalization for IBD flares as these represent the underlying high inflammatory states. The risk of VTE is highest during IBD-related surgery, non-intestinal surgery, and hospitalization due to flare-ups. The odds of VTE increased in a dose-dependent manner for each additional day of presurgical hospitalization [54, 55]. Patients who develop postoperative VTE are more likely to develop other postoperative complications and have a higher risk of death [56]. Therefore, current guidelines recommend VTE prophylaxis for all hospitalized IBD patients with low molecular weight heparin or fondaparinux over low-dose unfractionated heparin [57, 58]. In the perioperative period, prophylaxis should be continued throughout hospitalization.

Studies have shown that patients with IBD remain at an elevated risk of VTE within six weeks after surgery, likely secondary to subclinical hypercoagulability [59, 60]. Data from patients undergoing colorectal surgery for malignancy showed a 60% reduction in VTE at 90 days with the use of thromboprophylaxis for 30 days after hospital discharge [61]. Studies have also shown that the risk of VTE in patients with IBD following colectomy is similar, if not higher, than that in patients with colorectal cancer [62]. Although several risk stratification tools exist, they are not specific to the IBD surgical population, and prospective studies are needed to develop a model to identify patients who would benefit from prolonged postoperative thromboprophylaxis and to study its efficacy in patients with IBD [63].

## Management of Perioperative Immune Suppression

**Fig. 1:**
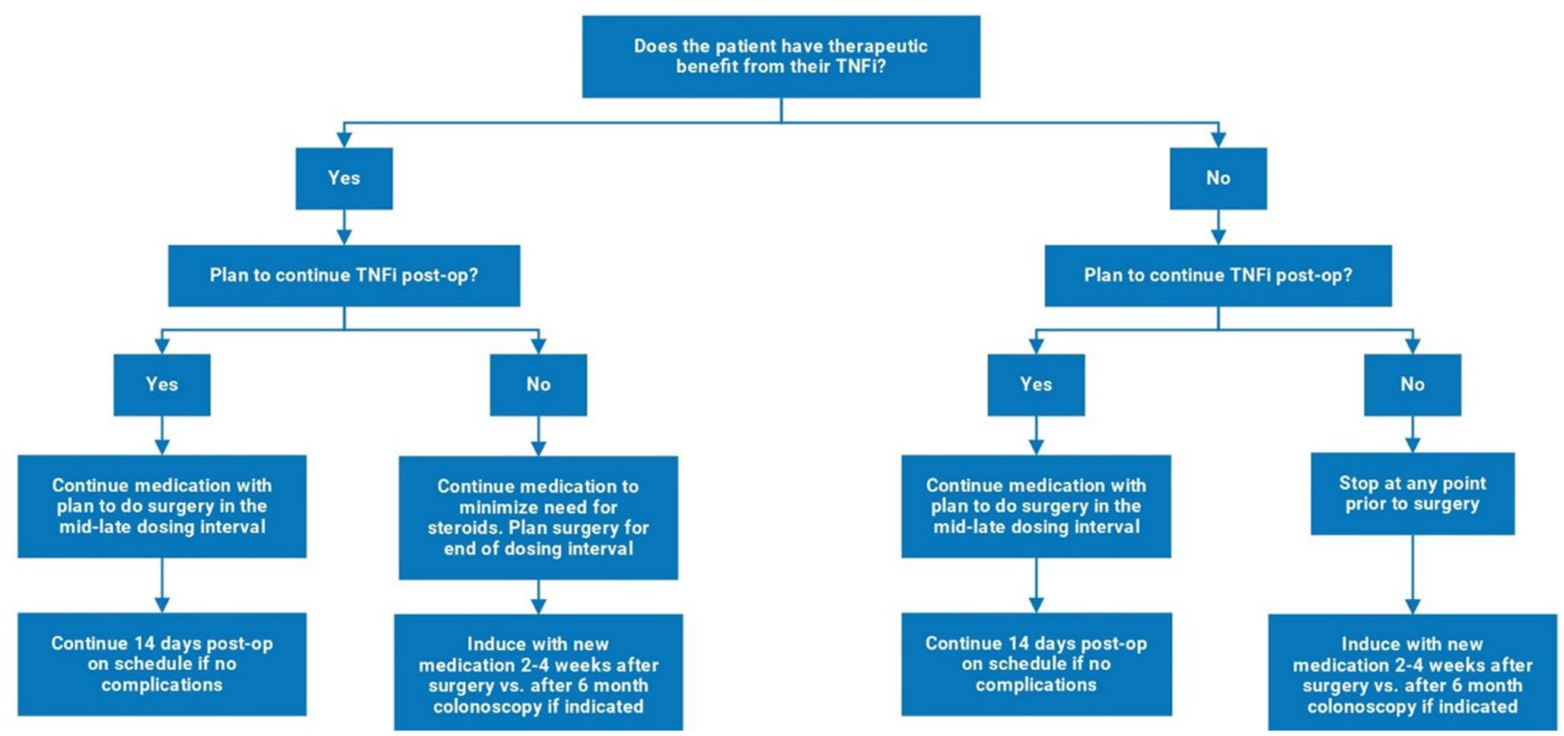
**Decision algorithm for management of Tumor Necrosis Factor inhibitors (TNFi) in the perioperative period**. The TNFi management plan will based on the current clinical benefit of the drug as well as the longterm plan for usage in the postoperative period. A similar decision algorithm can be applied for other biologic medications as available data does not suggest perioperative risk associated with them

The use of biologics, oral small-molecule drugs, and steroids is common in patients with Crohn’s disease; however, their use in the perioperative period remains controversial, as biologics have been postulated to be associated with an increased risk of postoperative infectious complications. Early retrospective studies and meta-analyses reported conflicting results. With the approval of new medications, there is a paucity of literature regarding their safety, further driving the uncertainty of their use in the perioperative period. The decision to continue immunosuppressive medications in the perioperative period depends not only on its safety and risk profile but also on whether the patient has a therapeutic benefit from the drug and if the same drug will be continued postoperatively (Fig. 1).

## Immunomodulators (6-mercaptopurine/Azathioprine and Methotrexate)

6-MP and azathioprine are immunomodulators (IMM) commonly used as steroid-sparing agents in patients with CD to maintain remission. They have a broad side effect profile, including hepatotoxicity, bone marrow suppression, pancreatitis, and leukopenia. Compared to patients not exposed to IMM, those who receive AZA or 6MP preoperatively do not have an increased risk of postoperative infectious complications [64, 65]. Based on current evidence, it is recommended to hold thiopurine on the day of surgery, and it is safe to resume these medications within 3 days of surgery or when other oral medications are resumed if renal function remains stable and there is an indication to continue the drug [66, 67]. 

Methotrexate (MTX) is also used as a steroid-sparing agent for patients with CD. The side effects include thrombocytopenia, hepatotoxicity, and pneumonitis. In the setting of renal impairment, its metabolites can lead to bone marrow suppression, which can occur during the perioperative period owing to an increased risk of infectious complications. A recent retrospective study examined patients with IBD and RA on MTX undergoing surgery and concluded that there was no increase in early postoperative complications with MTX use [68]. Data from the Prospective Cohort Study to Investigate the Safety of Preoperative Tumor Necrosis Factor Inhibitor Exposure in Patients With Inflammatory Bowel Disease Undergoing Intra-abdominal Surgery (PUCCINI)cohort study also did not show an increased risk of infectious or overall complications with preoperative use of MTX [65]. Therefore, we believe that it is safe to continue methotrexate in the preoperative and immediate postoperative periods, unless complicated by significant renal dysfunction or opportunistic infections.

## Steroids

Corticosteroids are widely used in the treatment of CD, particularly during acute flares and approximately 20–30% of patients become steroid-dependent during the course of their disease [69]. Corticosteroid use has been consistently identified as an independent risk factor for postoperative complications. A retrospective study showed that patients receiving corticosteroids preoperatively had 3.7 times more odds of developing any postoperative complications and 5.5X higher odds of developing major infectious complications [64]. Corticosteroids were an independent risk factor for any infection and surgical site infection in the PUCCINI cohort [65]. Similarly, a large study using the National Surgical Quality Improvement Program (NSQIP) database showed that preoperative steroid use for > 10 days was associated with a higher risk of postoperative complications including intra-abdominal infections, sepsis, and VTE [70]. Therefore, steroids should be tapered preoperatively, and the lowest effective dose to maintain remission should be used.

Prolonged use of steroids has the potential to suppress the hypothalamic-pituitary-adrenocortical axis (HPA) which leads to secondary adrenal insufficiency. Discontinuation of chronic steroids in the preoperative setting has been postulated to lead to adrenal crisis and shock secondary to surgical stress and has been a reason for the use of stress dose steroids prior to surgery for many years. A review of the literature has shown that this practice is not based on evidence; instead, glucocorticoid coverage should be determined by the dose and duration of preoperative steroid use in each patient [71]. A prospective randomized non-inferiority trial comparing high-dose and low-dose steroids used perioperatively in patients with IBD undergoing surgery showed no difference in the risk of hemodynamic instability and a trend towards more infections in the high-dose group [72]. 

Therefore, the use of stress-dose steroids should be decided on a case-by-case basis. Patients receiving ≤ 5 mg prednisone or any other dose for < 3 weeks were not at risk of adrenal insufficiency and did not need to be administered high-dose steroids preoperatively. For patients receiving higher doses of steroids for longer durations (> 4 months), corticotropin stimulation tests can be considered to diagnose true adrenal insufficiency. If diagnosed, tapering of steroids should be performed in consultation with an endocrinologist and these patients should be treated with stress dose steroids.

## Tumor Necrosis Factor Inhibitors

More than 30 studies have assessed the effects of perioperative tumor necrosis factor-α inhibitor (TNFi) use on postoperative infectious complications, but the results have been conflicting. Several studies have found an increased risk of infectious complications with the use of TNFi agents. However, most of these studies were retrospective, single-center studies with a confounding effect of concomitant immunosuppressive therapies such as steroids. The criteria for defining TNF inhibitor exposure have been heterogeneous across all studies, and despite our ability to easily assess serum TNFi levels, only four of these studies used drug levels as an exposure definition [73–77]. Several meta-analyses have also yielded conflicting results and are limited by the poor quality of most available studies on this topic [76, 78]. 

To date, four prospective cohorts have examined the risk factors for postoperative complications. GETAID, a French group, examined a prospective cohort of CD patients undergoing surgery and found that TNFi use within 12 weeks of surgery was associated with postoperative morbidity [79]. However, the GETAID cohort included only 24% TNFi-exposed patients; drug levels were not assessed, and no association was found between the proximity of the TNFi dose to surgery and postoperative morbidity. A smaller prospective cohort study, REMIND, reported no association between TNFi use and postoperative complications, including assessment of perioperative drug levels [80]. The largest of the studies was the PUCCINI study, a multicenter, prospective cohort study that looked at multiple patient factors, disease factors, preoperative medication exposures, and surgical factors to predict the risk of postoperative infections. TNFi exposure was defined as either patient-reported exposure within 12 weeks of surgery or separately as a detectable perioperative TNFi drug level. The study showed that neither patient-reported preoperative use of TNFi nor detectable serum TNFi levels were independent risk factors for any postoperative infection or surgical site infection. However, steroids, prior bowel resection, smoking, and diabetes were significant predictors of infection and surgical site infection [65]. Biologics before surgery were also studied using the NSQIP IBD Collaborative; in 4,578 patients, half of whom were on biologics, no association between biologics and postoperative infections, surgical site infections, or anastomotic leaks was observed [81].

Considering all the existing evidence, we conclude that the preoperative use of TNFi should not delay surgery in most patients. A proposed algorithm for patients undergoing elective surgery for CD is to first determine the therapeutic benefit and need for postoperative TNFi use. For patients who will need TNFi postoperatively, we suggest checking TNFi levels with a plan to perform surgery at mid-to-late intervals of dosing. Postoperatively, TNFi should be continued as scheduled, unless surgical complications occur. For patients who will not be treated with TNFi postoperatively, surgery should be planned considering other risk factors and for patient optimization (Figure 1). It is important to resume dosing on-time or start a new medication postoperatively, if indicated, by two weeks postoperatively if there are no complications as there is evidence that histologic recurrence may begin within the first month and medical therapy may be more effective if started early [[82, [83].

## Vedolizumab

Vedolizumab (VDZ) is a monoclonal antibody against a4b7 integrin which was approved by the FDA for the treatment of moderate to severe CD in 2014. Data regarding its safety in the perioperative period and the risk of postoperative complications are still emerging. Initial studies performed at the Mayo Clinic showed an increased risk of overall complications and surgical site infections in patients exposed to VDZ within 12 weeks of surgery [84]. However, these studies mainly included patients who underwent surgery between May 2014 and December 2015, immediately after the initial approval of VDZ, thus representing a group of patients who were medically refractory and likely to delay surgery for too long. More recent studies, including a Cochrane review, have not shown an association between VDZ use and postoperative infections and complications [78, 85, 86]. Additionally, detectable VDZ levels at the time of surgery, whether high or low, were not associated with postoperative morbidity, ileus, wound infection, leakage, or readmission [87–89]. Therefore, the timing of surgery should not be affected by the administration of vedolizumab.

## Interleukin 12/23 and 23 Inhibitors

Ustekinumab is a human monoclonal antibody against (IL)-12 and IL-23. Data regarding the association of ustekinumab with postoperative infection are even sparser, but thus far, there has not been a signal for postoperative complications, including in the recent Cochrane review and large retrospective studies [61, 86, 87]. Risankizumab is an IL-23 selective cytokine inhibitor with a similar mechanism and safety profile. Although no studies have evaluated the risk of postoperative complications, it appears to be safe and unlikely to be associated with an increased risk of infection.

## Janus Kinase Inhibitors

Upadacitinib is an orally administered selective JAK1 inhibitor that has recently been approved for use in Crohn’s disease. There are no data on its safety in the preoperative period, and studies must be conducted to evaluate its association with postoperative complications. Limited real-world data on tofacitinib use for off-label Crohn’s disease have not suggested a safety signal with respect to postoperative complications [90]. 

## Risk Stratification and Monitoring for Postoperative Recurrence

Surgical resection for CD is not curative, and up to 90% of patients can have endoscopic recurrence in the neoterminal ileum within 12 months of surgery [91], and up to 25% of these patients may require repeat resection within 5 years of surgery [92]. Therefore, it is imperative to identify patients at high risk of recurrence and monitor them closely. Although no risk score has been validated for the prediction of postoperative recurrence, many risk factors have been identified, including, but not limited to, penetrating disease phenotype, two or more prior CD-related surgeries, current smoking, age < 30 years, a short interval between diagnosis and surgery (< 10 years), and the presence of perianal disease [50, 93–95]. 

The decision to initiate early prophylactic therapy versus endoscopically guided therapy at 6–12 months varies from case to case and can be based on the presence or absence of established risk factors. Patients with multiple risk factors, deemed to be at moderate to high risk of recurrence, may be treated with advanced therapies such as prophylaxis against postoperative CD recurrence [92, 96–98]. A randomized trial on postoperative CD patients, the POCER trial, showed a significant reduction in the rate of endoscopic recurrence at 18 months in patients who underwent ileocolonoscopy at 6 months regardless of their risk status [99]. Therefore, regardless of the risk factors, all patients should undergo ileocolonoscopy with evaluation of the neoterminal ileum and Rutgeerts scoring at 6–12 months post-surgery. Additionally, the American Gastroenterological Association recently published guidance on the use of biomarkers for monitoring postoperative Crohn’s disease, advocating for monitoring of calprotectin with a value < 50 µg/g in the first year associated with a low risk of endoscopic disease activity in patients on postoperative pharmacologic prophylaxis, or at a low risk of postoperative recurrence [100].

## Conclusion

The management of patients with CD undergoing surgery is complex and multifaceted, necessitating an MDT approach. Analogous to the concept of prehabilitation employed in cancer patients, the identification and modification of presurgical risk factors play a pivotal role in enhancing both mortality and surgical outcomes. Prehabilitation has been employed in many cancer patients undergoing surgery and has been shown to improve quality of life, mortality, and morbidity [99]. Extending this concept to patients with CD undergoing surgery, in our opinion, the establishment of a dedicated prehabilitation clinic, involving all MDT members, emerges as a promising strategy at centers with high volumes and available resources. The primary objective of the clinic is to screen patients to identify potential risk factors such as smoking, malnutrition, intra-abdominal abscesses, and psychological stressors. Different members of the MDT can then intervene to address the identified risk factors by employing tailored strategies based on established recommendations. Subsequently, patients can be assessed in the clinic postoperatively to determine their risk status and initiate early postoperative prophylaxis if indicated (Table 2).

**Table 2:** Stages of perioperative management of CD

Stage I	Stage II	Stage III
Screening	**Prehabilitation**	Post Operative Care*
• Nutrition Status • Smoking • Physical Activity • Medications • Intra-abdominal Abscess • Psychological assessment	• Initiate EEN +/- TPN, treat anemia • Smoking Cessation Program • Physical Therapy Referral • Wean steroids, timing with anti-TNF • Drain/antibiotics • Referral to Psychologist	• Determine risk status based on presence or absence of risk factors • Early medication prophylaxis vs. ileocolonoscopy guided treatment • Fecal calprotectin assessment at 3–6 months post-op • If calprotectin elevated > 50 or patient is high-risk for recurrence: ileocolonoscopy at 6–12 months post-op 1,2

*Based on AGA Clinical Practice Guideline on the Role of Biomarkers for the AGA guidelines for Management of Crohn’s Disease and Management of Crohn’s disease after surgical resection

## Footnotes

1 Nguyen et al., “American Gastroenterological Association Institute Guideline on the Management of Crohn’s Disease After Surgical Resection.”

2 Ananthakrishnan et al., “AGA Clinical Practice Guideline on the Role of Biomarkers for the Management of Crohn’s Disease.”
